# A Mobile Health Intervention System for Women With Coronary Heart Disease: Usability Study

**DOI:** 10.2196/16420

**Published:** 2020-06-03

**Authors:** Avijit Sengupta, Theresa Beckie, Kaushik Dutta, Arup Dey, Sriram Chellappan

**Affiliations:** 1 Information Systems and Decision Sciences University of South Florida Tampa, FL United States; 2 College of Nursing University of South Florida Tampa, FL United States; 3 College of Engineering University of South Florida Tampa, FL United States

**Keywords:** coronary heart disease, mobile health technology, behavior change interventions, women, mobile phone

## Abstract

**Background:**

Coronary heart disease (CHD) is the leading cause of death and disability among American women. The prevalence of CHD is expected to increase by more than 40% by 2035. In 2015, the estimated cost of caring for patients with CHD was US $182 billion in the United States; hospitalizations accounted for more than half of the costs. Compared with men, women with CHD or those who have undergone coronary revascularization have up to 30% more rehospitalizations within 30 days and up to 1 year. Center-based cardiac rehabilitation is the gold standard of care after an acute coronary event, but few women attend these valuable programs. Effective home-based interventions for improving cardiovascular health among women with CHD are vital for addressing this gap in care.

**Objective:**

The ubiquity of mobile phones has made mobile health (mHealth) behavioral interventions a viable option to improve healthy behaviors of both women and men with CHD. First, this study aimed to examine the usability of a prototypic mHealth intervention designed specifically for women with CHD (herein referred to as HerBeat). Second, we examined the influence of HerBeat on selected health behaviors (self-efficacy for diet, exercise, and managing chronic illness) and psychological (perceived stress and depressive symptoms) characteristics of the participants.

**Methods:**

Using a single-group, pretest, posttest design, 10 women participated in the 12-week usability study. Participants were provided a smartphone and a smartwatch on which the HerBeat app was installed. Using a web portal dashboard, a health coach monitored participants’ ecological momentary assessment data, their behavioral data, and their heart rate and step count. Participants then completed a 12-week follow-up assessment.

**Results:**

All 10 women (age: mean 64.4 years, SD 6.3 years) completed the study. The usability and acceptability of HerBeat were good, with a mean system usability score of 83.60 (SD 16.3). The participants demonstrated statistically significant improvements in waist circumference (*P*=.048), weight (*P*=.02), and BMI (*P*=.01). Furthermore, depressive symptoms, measured with the Patient Health Questionnaire-9, significantly improved from baseline (*P*=.04).

**Conclusions:**

The mHealth prototype was feasible and usable for women with CHD. Participants provided data that were useful for further development of HerBeat. The mHealth intervention is expected to help women with CHD self-manage their health behaviors. A randomized controlled trial is needed to further verify the findings.

## Introduction

Center-based cardiac rehabilitation (CBCR) is a multidisciplinary, comprehensive, evidence-based intervention with proven morbidity and mortality benefits [1-4]. Outpatient CBCR in the United States generally takes place three times per week over 12 weeks [1,2]. Cardiac rehabilitation is the gold standard of care for the secondary prevention of cardiovascular (CV) disease and focuses on healthy behaviors, including physical activity (PA), healthy eating, psychosocial counseling for stress management, medication adherence, and smoking cessation [1,2]. Although CBCR provides irrefutable health benefits compared with usual care, significant underutilization and lack of access make CBCR programs beneficial only to the few who have health insurance and transportation to the facility [3,4].

CBCR referral is a health care quality performance metric [5,6], yet for three decades, only 10% to 20% of eligible women have attended CBCR, with up to a 56% dropout rate [7-18]. CBCR underutilization stems from numerous intrapersonal, interpersonal, logistical, programmatic, and health system barriers [19,20]. Inadequate health insurance and copayments of up to US $250 per session deter women from CBCR participation [21]. Socioeconomically deprived women who face transportation challenges, family or work obligations, depression, anxiety, or low social support are especially unable to use CBCR [22-27]. These limitations have prompted a call to redesign CBCR for women [7,28,29].

Home-based cardiac rehabilitation (HBCR) offers a potential solution as it avoids conflicts with competing demands of daily life; however, limited evidence exists that HBCR is effective and will reach more women [30-32]. Our study is a direct response to the call to action to expand the reach of secondary prevention to women unable to attend CBCR [7]. We explore the feasibility of delivering technology supported behavior change interventions to women with coronary heart disease (CHD). On the basis of our previous proof-of-concept research [33-35], we translated our gender-specific, motivationally enhanced CBCR program to a prototype of a mobile health (mHealth) home-based behavioral intervention (referred to here as HerBeat). HerBeat has the potential to improve health behaviors and CV risk factors in women with CHD by overcoming barriers inherent in CBCR, expanding reach to the majority of women without access to CBCR, and integrating a home-based program seamlessly into their lives.

Up to 80% of CHD events are attributed to unhealthy behaviors [36]; adherence to health behaviors unquestionably improves CV health [8,36,37]. Fortunately, CBCR practice standards are widely disseminated and readily adaptable for a gender-specific HBCR, based on the results from HBCR studies [38,39]. CBCR-eligible patients given the choice between HBCR and CBCR are up to four times more likely to participate in HBCR [40-42]. Compared with CBCR, HBCR overcomes logistical barriers to access, the need for expensive facilities, specialized exercise equipment, and high personnel costs and provides education, coaching, and monitoring by a health coach through, when available, wearable sensors and smartphones that are potentially operational 24 hours a day, 7 days a week [20,43]. Moreover, HBCR assesses daily PA, whereas CBCR only measures supervised exercise sessions [44]. Most CHD patients spend over 5000 waking hours yearly, independent of medical providers [45], and thus, arming them with behavior change techniques (BCTs) that can be implemented anytime is crucial.

A BCT is defined as an observable and replicable intervention component designed to redirect causal processes that regulate behavior, a technique proposed to be an active ingredient [46]. Unlike most mHealth interventions that deliver text messages at preset times, largely unrelated to patient behavior [47], HerBeat delivers personalized, just-in-time adaptive interventions comprising gender-specific, behavior theory–based BCTs in response to proximal behaviors and moods. Theoretically derived BCTs delivered anytime and anywhere are essential to forming and maintaining health behaviors into lifelong habits. We used four specific BCTs as we designed interventions to be deployed through HerBeat: (1) goals and planning, (2) feedback and monitoring, (3) shaping knowledge, and (4) repetition and substitution [46]. For the BCT goals and planning, we used the subtechniques *goal setting* and *review behavior goal* for creating instantiations of the intervention. For the BCT feedback and monitoring, we used subtechniques such as *feedback on behavior*, *self-monitoring of behavior*, *monitoring of outcomes*, and *feedback on outcomes*. We used subtechniques such as *instructions on how to perform the behavior* and *information about antecedents* for the BCT shaping knowledge, and for the BCT repetition and substitution, we used subtechniques such as *graded tasks* and *habit formation and habit reversal*. Higher levels of self-monitoring/management and unsupervised exercise inherent in HBCR vs CBCR can aid transition from active intervention to lifelong self-management seamlessly.

First, the purpose of this study was to examine the usability of a prototype of HerBeat for women with CHD. Second, we sought to examine the potential influence of the prototype on health behaviors (eating habits, PA, and goal setting) and psychosocial characteristics (self-efficacy [SE], depressive symptoms, and perceived stress) at the 12-week follow-up visit.

## Methods

### Design Overview

We used a smartwatch app and a smartphone app to collect data on a patient’s daily PA, heart rate, eating episodes, and mood. Data from the sensors embedded in the smartwatch are interpreted as step counts and heart rate and are sent to the smartphone via Bluetooth and then to a cloud drive via Wi-Fi or 4G. All data uploaded to the cloud are then downloaded immediately and uploaded to a server over a secured virtual private network connection through the public internet. Data in the server are analyzed and projected on the dashboard for the health coach to view. The old data are archived and then refreshed by the most recent data on the dashboard every 10 min.

### Intervention

**Figure 1:**
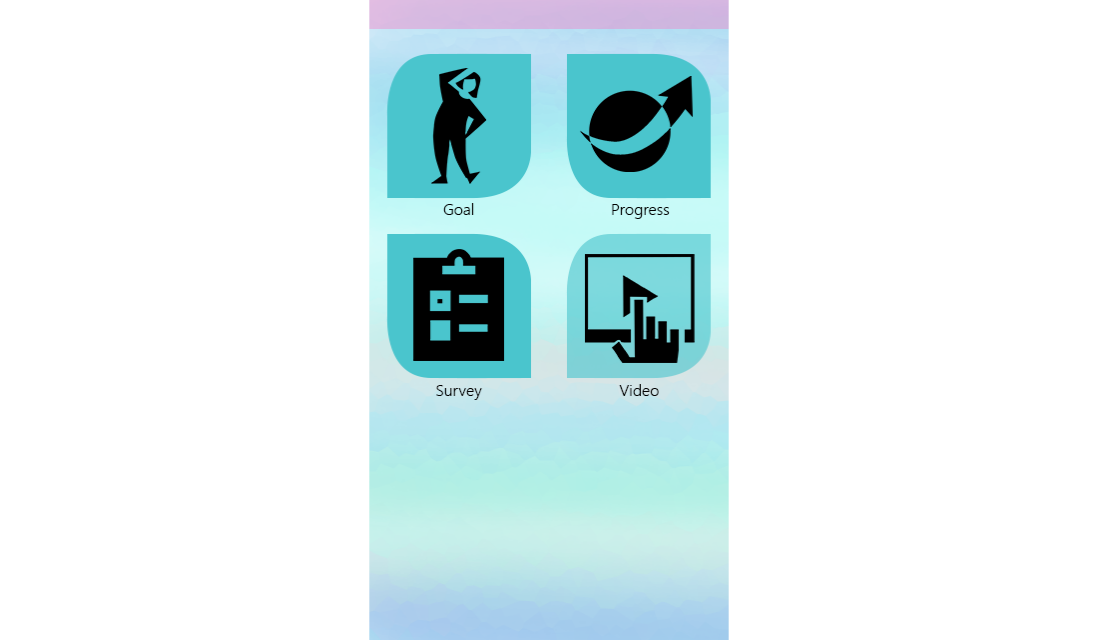
Main menu screen of HerBeat application.

The HerBeat prototype included a wrist-worn smartwatch (Moto 360 2nd Gen, Android Wear OS 2.0) and a smartphone (Samsung Galaxy S6, Android 7.0), with the app installed on both devices and a web-based dashboard for monitoring participant data. The 4 features of the prototype included (1) goal setting, (2) progress, (3) ecological momentary assessment (EMA) surveys, and (4) videos (see Figure 1). The goal setting feature allowed participants to set multiple walking goals for up to 60 min each. Study participants were tasked with setting their own PA goals in terms of the number of minutes walked. Participants were also prompted to report their readiness to begin PA and their current level of energy on a scale of 1 to 10. After setting a PA goal, each participant was sent a motivational message that encouraged exercise. Data about the participant goal setting and subsequent PA performance were monitored through a web-based dashboard in real time by a trained professional.

**Figure 2:**
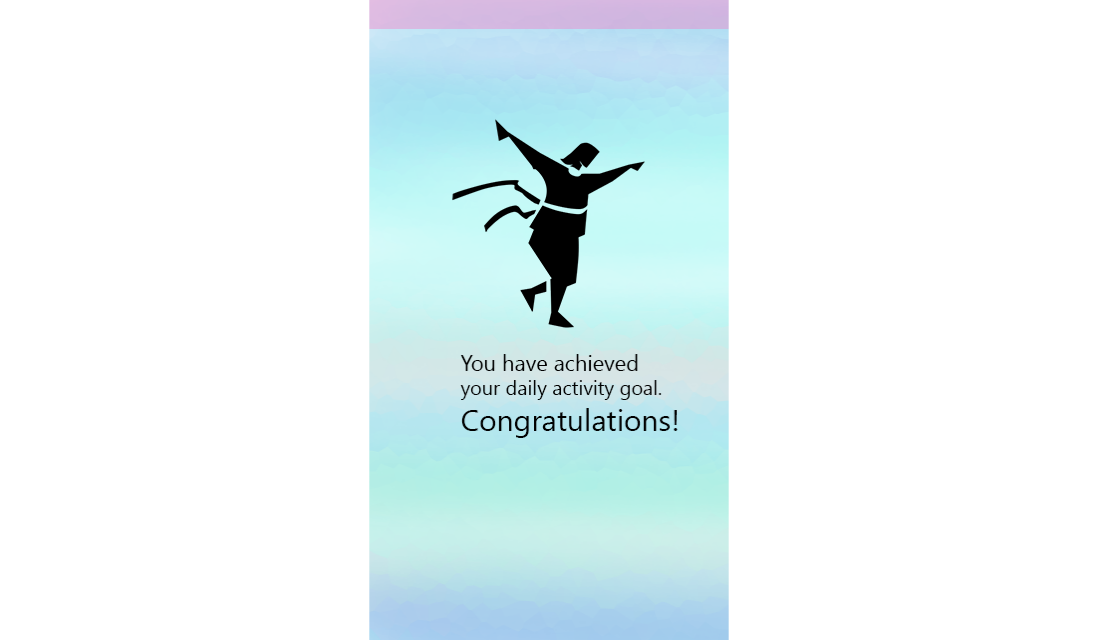
Example of graphic user interfaces of interventions (congratulatory message for achieving goal).

**Figure 3:**
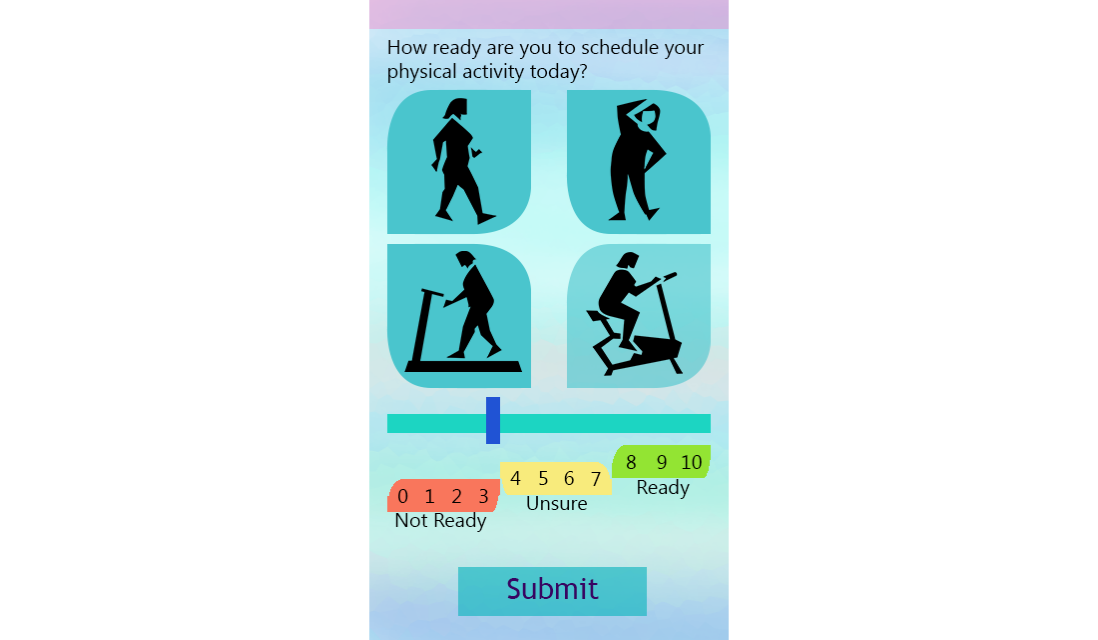
Example of graphic user interfaces of interventions (physical activity schedule).

**Figure 4:**
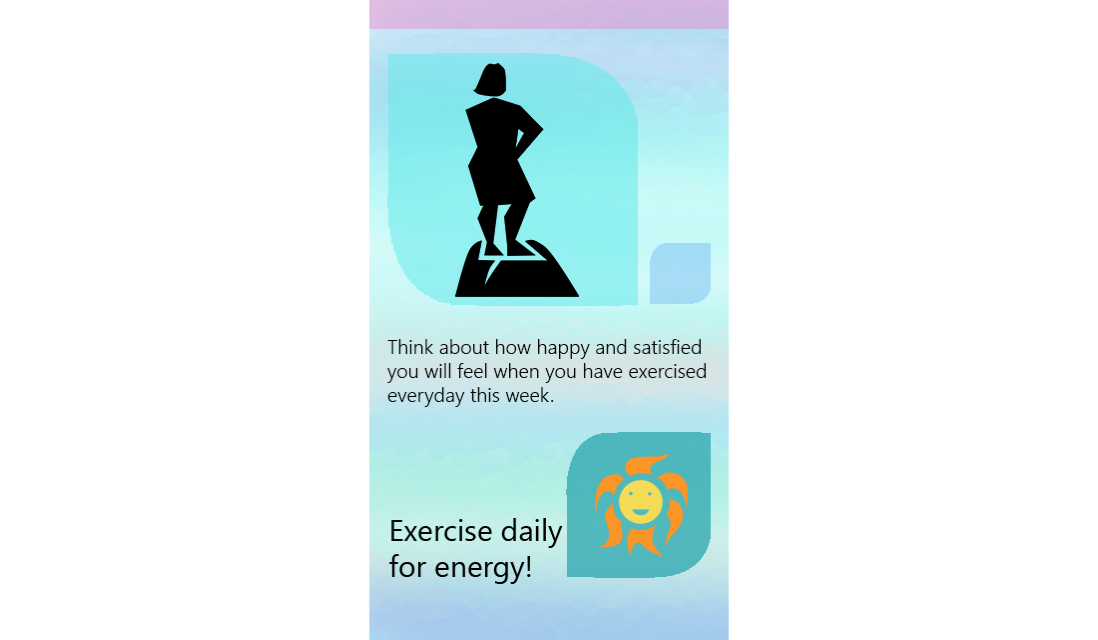
Example of graphic user interfaces of interventions (prompting to exercise).

**Figure 5:**
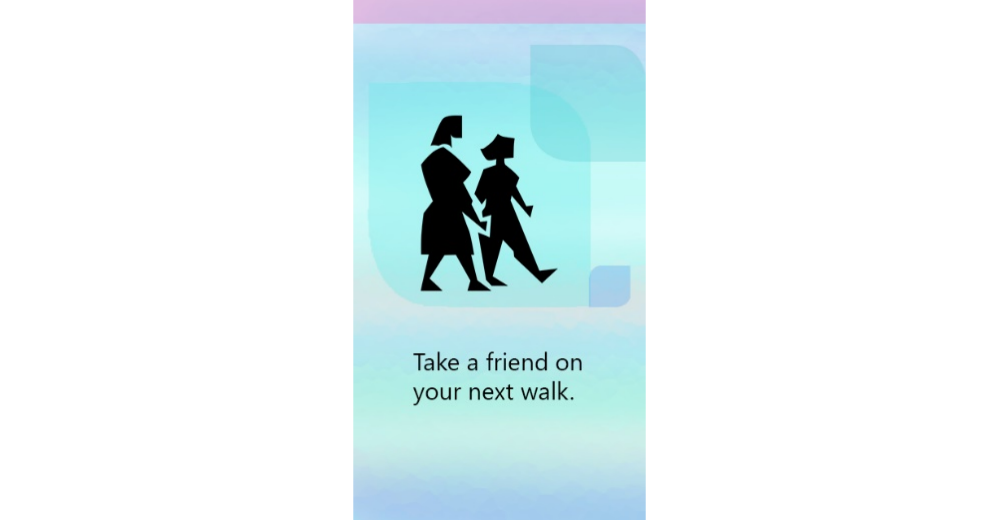
Example of graphic user interfaces of interventions (positive reinforcing message).

The progress function permitted participants to review the number of minutes walked, number of steps taken, and distance covered in miles. If participants had not completed their goal when seeking progress, they were presented with the number of minutes remaining to goal completion. If a goal was completed, the participant was sent a gender-specific graphic user interface (GUI) with a congratulatory message for achieving their goal. The EMAs are described in the Measurement section. The final feature provided participants access to 9 customized short videos, developed by the principal investigator (PI) with expertise in behavioral medicine and women’s CV health, on healthy eating behavior and on guidelines for safe PA. The app also sent two types of behavior change intervention messages. If the participant had not set a PA goal by 4 PM daily, a message prompting them to exercise was sent. If participants were proactive in setting and achieving walking goals, they were sent a positive reinforcing message. Figures 2-5 show some of the examples of GUIs of intervention screens. The dashboard was used by the health coach to remotely monitor participants’ PA (step count), heart rate, goal setting behavior, responses to the EMA surveys, and frequency of accessing the health videos. The health coach, via the dashboard, also monitored episodes of Wi-Fi and Bluetooth disconnections. The health coach sent a personalized, encouraging message to engage with HerBeat to the participant’s smartphone about once a week.

### Recruitment

After obtaining approval from the university institutional review board and using a single-group, pretest, posttest design, we recruited participants from a university-affiliated outpatient cardiology clinic between May 2018 and August 2018. The cardiology clinic is part of an academic medical center and is staffed by faculty members who are physicians in the Division of Cardiovascular Sciences. The clinic provides state-of-the-art services and treatment options. Participants were recruited by the PI (health coach), who had access to the clinic’s electronic health record system. Potential participants who were scheduled to see their health care provider were approached for inclusion in the study after they had completed their clinic visit. Women were eligible for the study if they were aged 50 years or older; diagnosed with an acute coronary syndrome or coronary revascularization in the last 10 years; able to read, speak, and understand English; and able to participate in a PA, such as walking, unaided. We also sought verbal clearance from their cardiologist to participate in the study. Study exclusion criteria included residing outside a 50-mile radius of the study site; a psychiatric condition including dementia, delirium, or schizophrenia or actively undergoing acute psychiatric treatment; prior neurological brain disorders; current use of illicit drugs and/or chronic alcohol use at the discretion of the PI; or life-limiting comorbid conditions (eg, metastatic cancer).

### Study Procedures

The informed consent form clearly explained that study participation was voluntary and participants could withdraw from the study at any time without jeopardizing their health care. Their decision to withdraw had no impact on their relationship with their cardiologist. If they wished to withdraw study participation, they needed only to inform the PI, and no further data would be collected from that time onward.

After baseline assessment was completed, the participants were trained by a graduate student with technical expertise to use the smartwatch and the smartphone that were provided for the duration of the study. Technical questions were answered by one of the study personnel via telephone or in person. The participants were then asked to use the prototype for 12 weeks and return for a follow-up visit when data collection was completed and the hardware was returned. We did not explicitly request that participants improve their health behaviors because our primary focus was the usability of the HerBeat prototype.

Data collected from self-report questionnaires and physical assessments were maintained in a database using Research Electronic Data Capture software. The smartwatch streamed step count and heart rate data continuously between 6 AM and 10 PM daily every 3 min via Bluetooth and Wi-Fi to a Health Insurance Portability and Accountability Act–compliant server. We archived participants’ data by a study identification number to protect their identity. Daily backend jobs processing all collected information were automated on the server side. The resulting information was stored in a Structured Query Language (SQL) format for easy retrieval. The web portal dashboard was created from the SQL data to present the data to the health coach.

### Measures

#### Usability

We evaluated the participants’ perceptions of usefulness, ease of use, and satisfaction with HerBeat with the System Usability Scale (SUS) [48]. The SUS was first introduced in 1986 and consists of 10 items. The study by Lewis and Sauro [48] suggested that SUS has two different factors. The first factor consists of 8 items on a 5-point scale that measures how usable the system is, and the second factor measures how easy it is to learn the system. These correlated factors have reasonable reliability (coefficient α of .91 and .70, respectively) and correlated highly with the overall SUS [48]. A sensitivity analysis conducted by Lewis and Sauro [48] suggested that using data from 19 tests had a significant *test* by *scale* interaction, providing additional evidence of the differential utility of the scale. The SUS is frequently used by both researchers and practitioners, given the adequate reliability data and ease of implementation. Scoring guidelines of the SUS recommend transforming the scale to a 0 to 100 range. The SUS yields a single number representing a composite measure of overall usability, and scores for individual items have very limited meaning on their own. SUS follows a specific rubric and reverse scoring of certain items to calculate the final usability composite score from the scores against individual items.

#### Sociodemographic and Health History

At baseline, we collected data on cardiac history, comorbidities, medications, and CV risk factors as well as sociodemographic attributes, including age, marital status, work status, education, occupation, living arrangements, insurance status, and income.

#### Psychosocial

Dietary SE was measured using the 20-item Eating Habits Confidence Survey consisting of a 5-point scale ranging from 1 to 5, with higher scores reflecting higher SE [49]. This instrument has shown strong internal consistency reliability in overweight postmenopausal women [50]. Exercise SE was measured with the 12-item Exercise Confidence Survey asking participants to rate their confidence in maintaining an exercise routine when facing various barriers. Scores range from 12 to 60; higher scores reflect higher SE [49]. Participants’ perceptions of their SE for managing chronic illness were assessed with a 6-item instrument, with scores ranging from 6 to 60 [51]. The scale measures the perceived adaptability of survey participants to manage different aspects of chronic diseases, such as pain and fatigue, and the scores demonstrate good reliability (Cronbach α coefficient .89) [51]. The Perceived Stress Scale (PSS) [52] consists of 14 items that are measured on a 5-point scale, with higher scores reflecting greater perceived stress. PSS scores are obtained by first reversing the scores on the 7 positive items and then summing across all 14 items. The coefficient α reliability for the PSS was .85, and the validity of PSS was established by showing substantial correlations between the scale and standard symptomatology measures [52]. Depressive symptoms were measured with the Patient Health Questionnaire-9 (PHQ-9), with scores ranging from 0 to 17, with higher scores reflecting more depressive symptoms [53]. Scores from the PHQ-9 questionnaire items showed strong reliability and validity when used by researchers to measure major depressive disorder [54], depression [55] (Cronbach α .85), and depression in patients with CHD (Cronbach α .90) [56].

#### Behavioral

Eating behavior was assessed using the 13-item Rapid Eating Assessment for Participants-Short Form (REAP-S) [57]. Possible scores range from 13 to 39, with a higher score indicating better diet quality [58]. Self-reported PA was assessed using the 7-day recall International Physical Activity Questionnaire-Short Form (IPAQ-SF), which measures PA intensity, frequency, and duration [59]. Items in the IPAQ-SF were structured to provide separate scores on walking and moderate- and vigorous-intensity activities. The IPAQ-SF questionnaire showed moderate to strong reliability (intraclass correlation coefficient [ICC]) in prior research conducted with college students (ICC=0.71-0.89) [60], Chinese youth (ICC=0.43-0.83) [61], pregnant women (ICC=0.81-0.84) [62], and individuals with schizophrenia [63]. Step count and distance walked were measured objectively with the Moto 360 smartwatch over 12 weeks. The smartwatch determines step count via processing readings from the accelerometer and gyroscope sensor, from which we estimate the distance walked. The heart rate was collected continuously when the watch was worn. The smartwatch was worn every day, except while bathing, sleeping, or swimming.

#### Physiological

With participants in light clothing without shoes, weight was measured to the nearest 0.1 kg using research precision–grade, calibrated, digital scales, and height was measured to the nearest 0.1 cm using a freestanding stadiometer. BMI was calculated as weight (kg)/height (m^2^). Waist circumference, assessed just above the uppermost lateral border of the right ilium using a Gulick tape measure, was calculated to the nearest 0.1 cm as the mean of the second and third measures [64,65]. Blood pressure (BP) was obtained with a calibrated automated monitor according to the standard protocol [66].

#### Ecological Momentary Assessments

Participants completed brief 1- to 2-min surveys sent to their smartphone at 8 random times throughout the day. These surveys asked about their current activity, location, mood, eating episodes, and who they were with.

### Data Analysis

Data analysis techniques are applied to gain insights into the patient’s activity, heart rate, and EMA survey response data. Patients’ physical activities and EMA responses are analyzed through a decision rule-based expert system as well as by the health coach. These data are used to send standard preprogrammed intervention messages to the patients by the system and to help the health coach to customize intervention messages to send to patients through the dashboard at the right time to maximize their impact.

Descriptive statistics (eg, univariate graphical and numerical statistics, bivariate distributions, scatterplots, and counts/percentages) were generated and summarized for all study data. Paired *t* tests were used to compare continuously measured variables from baseline to the 12-week posttest measures. Given the small sample size, we were generally underpowered to perform parametric statistics. The α level was set at *P*≤.05. Qualitative field notes were summarized across all participants for themes.

## Results

### Participants

A total of 11 participants signed the informed consent form, and 10 participants completed data collection. Of the 10 participants, 2, both recently experiencing traumatic life events, engaged very little with HerBeat. Most of the participants were white (8/10, 80%), married, or partnered (6/10, 60%) women with a mean age of 64 years (range 53-75 years; SD 6 years; see Table 1). The majority of participants had health insurance and an income of at least US $40,000 annually; 5 participants worked full time. All participants had CHD, with 2 participants diagnosed with a myocardial infarction and one with heart failure. Moreover, 50% (5/10) of participants had undergone a percutaneous coronary intervention, and 20% (2/10) of participants had undergone a coronary artery bypass graft surgery. None of the participants had ever attended a CBCR program.

The participants had multiple comorbidities including diabetes mellitus (3/10, 30%), osteoarthritis (4/10, 40%), and orthopedic disorders (2/10, 20%), and one participant was being treated for skin cancer (1/10, 10%; see Table 2). The participants exhibited traditional CV disease risk factors, including dyslipidemia, hypertension, physical inactivity, familial heart disease, and being overweight. Most participants had never used tobacco; former smokers had a mean of 23.75 (SD 19.3) pack-years of smoking. Participants were prescribed numerous evidence-based CV medications to treat their chronic conditions.

Table 3 summarizes the baseline and 12-week follow-up physiological and psychosocial participant characteristics. Although we observed no changes in BP, the participants had statistically significant improvements in waist circumference (*P*=.048), weight (*P*=.02), and BMI (*P*=.01). Furthermore, participants’ depressive symptoms significantly improved from baseline (*P*=.04).

SE for exercise, diet, and managing chronic illness was not statistically significantly different from baseline, although it trended in the desired direction. Participants also demonstrated nonsignificant improvements in REAP-S scores and perceived stress.

**Table 1:** Participants’ sociodemographic data (n=10).

Characteristics		Values
**Age (years)**		
	Mean (SD)	64.4 (6.3)
	Range	53-75
**Race or ethnicity, n (%)**		
	White	8 (80)
	Black, African American	1 (10)
	Asian/Pacific Islander	1 (10)
**Education, n (%)**		
	Community college	5 (50)
	4-year college incomplete	1 (10)
	4-year degree	1 (10)
	Master’s degree	2 (20)
	Doctoral degree	1 (10)
**Employment status, n (%)**		
	Employed full time	5 (50)
	Not employed or retired	5 (50)
**Marital status, n (%)**		
	Married/partnered	6 (60)
	Divorced	2 (20)
	Widowed	2 (20)
**Primary insurance status, n (%)**		
	Private insurance	6 (60)
	TriCare (military/veterans)	1 (10)
	Medicaid	2 (20)
	Medicare	1 (10)
**Annual household income (US $), n (%)**		
	20,000 to <40,000	3 (30)
	40,000 to <80,000	2 (20)
	80,000 to <100,000	2 (20)
	≥100,000	3 (30)

**Table 2:** Clinical characteristics of participants (n=10).

Characteristics		Value, n (%)	
**Cardiovascular disease diagnosis**			
	Coronary heart disease		8 (80)
	Myocardial infarction		1 (10)
	Congestive heart failure		1 (10)
**Comorbidities**			
	Diabetes		3 (30)
	Arthritis		4 (40)
	Orthopedic disorder		2 (20)
	Skin cancer		1 (10)
**Cardiovascular risk factors**			
	Overweight (BMI 25.0-29.9 kg/m^2^) or obese (BMI >30 kg/m^2^)		6 (60)
	Familial heart disease (onset before 60 years and 50 years in mother and father, respectively)		2 (20)
	Dyslipidemia		10 (100)
	Hypertension		6 (60)
	Physical inactivity (<30 min 5 times weekly)		8 (80)
**Tobacco use**			
	Never		4 (40)
	Former		6 (60)
**Medication classes prescribed**			
	Beta blocker		8 (80)
	Calcium channel blocker		2 (20)
	Angiotensin-converting enzyme inhibitor		4 (40)
	Angiotensin receptor blocker		3 (30)
	Statin		10 (100)
	Insulin		2 (20)
	Metformin		2 (20)
	Aspirin		9 (90)
	Clopidogrel		5 (50)
	Other antiplatelet		3 (30)

**Table 3:** Physiological and psychosocial characteristics (n=10).

Characteristics^a^		Baseline, mean (SD)	12-week follow-up, mean (SD)	*P* value
Systolic blood pressure (mm Hg)		129.2 (12.3)	141.5 (18.9)	NS^b^
Diastolic blood pressure (mm Hg)		76.7 (8.7)	73.6 (9.2)	NS
Waist (cm)		97.7 (14.7)	95.4 (12.6)	.048
Weight (kg)		80.5 (19.7)	79.1 (18.6)	.02
BMI (kg/m^2^)		29.2 (6.0)	28.7 (5.8)	.01
Self-Efficacy Scale for Managing Chronic Disease		45.4 (12.5)	48.2 (7.6)	NS
Self-efficacy for exercise behavior		52.5 (7.6)	54.4 (6.2)	NS
Self-efficacy for diet		88.8 (6.0)	89.6 (6.8)	NS
Perceived Stress Scale		13.3 (6.7)	9.9 (6.9)	NS
Patient Health Questionnaire-9		5.5 (5.4)	2.9 (3.8)	.04
Rapid Eating Assessment for Participants-Short Form		32.7 (3.5)	33.7 (2.7)	NS
**International Physical Activity Questionnaire (last 7 days)**				
	Days of moderate physical activity	3.0 (2.4)	3.4 (2.3)	NS
	Minutes per day of moderate physical activity	35.7 (35.3)	63.1 (52.8)	NS
	Minutes sitting on 1 week day	330.0 (124.1)	331.0 (212.6)	NS
	Days walked at least 10 min per day	5.4 (2.3)	5.5 (1.7)	NS
System Usability Scale		N/A^c^	83.6 (16.4)	N/A

^a^α≤.05.

^b^NS: not significant.

^c^N/A: not applicable.

### Engagement With the Prototype

Over the course of the study, participants (n=8) collectively set 132 goals, with a mean of 16.5 (SD 17.3) goals per participant for a collective total of 3335 min of walking, with a mean of 34.72 (SD 41.68) min per participant (see Table 4) per week. Most of the walking goals were set between 9 AM and 11 AM and 5 PM and 6 PM. Over the course of the study, smartwatches allocated to the participants collectively recorded 4933 min of walking, with a mean of 22.02 (SD 35.32) min per participant per day. That is, the participants walked more than they intended when setting a goal. Over 12 weeks, each participant walked a mean of 28 days (out of a possible 90 days) and took a mean of 3718.8 (SD 3826.0) steps per day. The group responded to 830 EMA surveys and accessed 8 health educational videos 165 times during the study. The participants accessed more videos related to healthy eating behavior (137/165, 83%) than those related to PA (28/165, 17%). The participants received a total of 265 automated intervention messages based on their progress toward their goals.

**Table 4:** Participants’ engagement (N=8).

HerBeat features		Value, range	Value, mean (SD)
**Goals**			
	Number of goals set per participant	3-52	16.5 (17.3)
	Walking goal set (minutes) per participant per week	1-60	34.72 (41.68)
**Progress**			
	Daily walking (minutes)	1-132	22.02 (35.32)
	Daily steps per participant	3-21,179	3718.8 (3826.0)
	Daily miles per participant	0.1-10.6	1.86 (1.9)
**Videos**			
	Number of times health videos were accessed per participant per week	0-17	1.96 (1.76)
	Number of ecological momentary assessment survey responses per participant per week	0-36	8.64 (9.45)
	Behavior change messages acknowledged per participant per week	0-7	2.75 (2.65)

### Usability

The mean score on the SUS was 83.60 (SD 16.4). Participants generally found HerBeat to be easy to learn and use. They also found the functionalities to be well integrated, and they felt confident in using HerBeat. The participants did not find it unnecessarily complex or cumbersome to use (see Table 5).

**Table 5:** Descriptive statistics of the System Usability Scale items.

No	Item	Value, mean (SD)
1	I think I would like to use this system frequently.	79.5 (2.13)
2	I found the product unnecessarily complex. (R)^a^	83.5 (1.15)
3	I thought the product was easy to use.	86.6 (1.29)
4	I think that I would need the support of a technical person to be able to use this product. (R)	91.0 (2.25)
5	I found that the various functions in this product were well integrated.	83.1 (1.79)
6	I thought that there was too much inconsistency in this product. (R)	81.1 (1.26)
7	I would imagine that most people would learn to use this product very quickly.	82.2 (1.28)
8	I found the product very cumbersome to use. (R)	78.7 (2.14)
9	I felt very confident using the product.	87.7 (2.17)
10	I needed to learn a lot of things before I could get going with this product.	82.2 (1.02)

^a^(R)=reversed scored item.

They also reported not requiring the support of a technical person to use HerBeat. Only one patient required a home visit to address a technical issue. Participants’ themes derived from field notes mostly involved technical issues. The most frequent complaint was the short battery life of the smartwatch. We rectified this problem after valuable participants’ input. Some working participants found it difficult to carry both a personal phone and a study phone and respond to EMA surveys during the day. A participant who worked in a library sought permission from her supervisor to carry the study phone and respond to the EMA surveys. One participant requested taking HerBeat with her to Europe to allow her to track her activity while on vacation.

Participants’ feedback also led to the redesign of some of the GUIs of the EMA survey. Although there was minimal contact between the health coach and the participants during the 12 weeks and participants went on vacation during the study, they voiced reassurance that their progress was being monitored by the health coach via the dashboard. Participants had no adverse events during the study, and there were no issues raised about privacy concerns.

Data captured during our study suggest that at least one of the participants set a walking goal of 1 min and at least one of the participants watched no health-related videos during the study. We probed the corresponding participants during the final debriefing session about these data. For the first observation, the participant suggested that the walking goal of 1 min was mistakenly set while exploring the goal setting function at the very beginning of the study. The participant’s intention was to navigate further inside the goal setting function. Regarding the second observation, the participant chose not to watch any health-related videos because she felt well informed about these health behaviors.

## Discussion

### Principal Findings

The primary aim of this study was to determine the usability of our mHealth system, HerBeat, with a cohort of women with CHD before proceeding with the development of a comprehensive home-based secondary prevention intervention. Our secondary aim was to evaluate the influence of HerBeat on various psychosocial and health behaviors of the participants. To our knowledge, this is the first study to evaluate the usability of a gender-specific mHealth app for secondary prevention of CHD in women. The main finding of the study was that the system was acceptable and usable in its prototypic form. The level of engagement of participants with HerBeat was greater than anticipated, given the relatively primitive features. We developed HerBeat to avoid high data entry burden and designed gender-specific GUIs to foster engagement. Given that 80% of health-related apps are abandoned after only 2 weeks [67], the engagement of the participants with our prototype was good, particularly when they were given little prodding for using the technology. We viewed this as an encouragement to proceed with the expanded version of HerBeat, with increased involvement of the health coach.

### Additional Findings

Comparisons of user engagement with mobile apps of participants with characteristics similar to the participants in our study are difficult to make because usability was defined differently in these studies [68-71]. Some described metrics such as app usage frequency, duration, data registration, or responsiveness of the user to daily tasks. In addition to the often low participant numbers, dropouts, and short study duration, conclusions about engagement are difficult to draw. Completion of tasks within the app, such as completion of an education module, was a typical measure of use in studies with a focus on healthy lifestyle. Forman et al [68] gauged engagement by patient completion of at least one prescribed daily task. In other studies, emphasis was placed on logging medication intake or physical measurements [69,70]. The authors did not report the acceptability of a data entry requirement. We made the decision early during development, based on numerous interviews with patients, to avoid the requirement of data entry to reduce respondent burden. In an uncontrolled single-group, pretest, posttest design [72], participants were required to log daily BP measurements for 55 days; however, it was unclear whether all patients logged BP on each of the 55 days. Patients in one small study of both heart failure and CHD participants [73] appreciated medication reminders and PA information. However, they felt that daily requirements for data entry or other responses were inconvenient. Clearly, high data entry burden is a usability issue [74].

We did not see evidence of the message fatigue reported by others [75]. The fact that the participants responded to 830 EMA surveys over 12 weeks was, in our opinion, quite remarkable. Although the number of EMA survey responses was greater than expected, the responses declined over time. Educational videos on healthy eating behavior were viewed more often than videos related to physical activities, presumably because eating a healthy diet is often a daily or hourly struggle between reflex and self-control. Participants may have viewed the videos to seek assistance with making healthy eating decisions. Eating and body weight regulation is a complex process that involves both metabolic and hormonal control mechanisms and neurocognitive processes involved with memories, expectations, and evaluation of food and the consequences of eating [76]. The decisions about what and when to eat are a balance between reflexive behavior and higher-level cognitive processes. Eating can be reflexive and automatic by the mere smell of a favored food [77]. This reflexive eating can be opposed by dietary restraint of choosing a healthy food that involves higher-level cognitive processes to counter the power of tempting environmental stimuli [78].

On the basis of decision rules related to participants’ responses employed in HerBeat, some intervention messages were deployed more frequently than others. Most participants exceeded the walking goals they set. In other words, most of the time participants did not abruptly stop their walk after achieving their PA goal but rather exercised beyond the goal. We hypothesize that this may reflect low SE when setting the goal, followed by greater confidence when they surpassed the goal. Although we did not set a target for time spent walking or for step count, the participants’ daily step count was relatively modest. A common goal of 10,000 steps per day has been perpetuated by the lay press and is often used as the default by software programs on wearables and smartphones [79]. In the United States, the average number of steps accrued daily (measured by smartphones) is approximately 4800; worldwide, it is approximately 5000 [80]. There is sparse data on the number of daily steps needed for health [81,82] or clinical outcomes and mortality [83]. In the Women’s Health Study, a cohort of 16,741 women with a mean age of 72 years wore accelerometers to measure their steps per day over 7 days [84]. Women who averaged 4400 steps per day had significantly lower mortality rates during a follow-up of 4.3 years compared with the least active women who took approximately 2700 steps per day. As more steps per day were accrued, mortality rates progressively decreased before leveling at approximately 7500 steps per day [84].

Although we did not expect participant health behaviors, SE, perceived stress, or depressive symptoms to improve with a limited functionality prototype, we nonetheless observed significant reductions in waist circumference, weight, and BMI as well as reduced depressive symptoms after study participation. These improvements were unexpected because the research team had minimal contact with the participants during the 12 weeks, and we did not prompt them to set goals for walking. Participants reported minimal positive changes in their SE for exercise, diet, or managing chronic illness, but scores nonetheless trended in the expected direction. From baseline to the 12-week follow-up, there was a modest increase in the mean minutes of moderate-intensity exercise. There were no reductions in participants’ time spent sitting. The primary purpose of this study was to examine the usability of the system, and secondarily, to examine behavior change after the 12-week study. With a more robust version of the system, we will examine the effectiveness of the system in a randomized clinical trial.

### Limitations

Our findings must be balanced with the limitations of the study. First, this was a small convenience sample from a single study site. With multiple statistical testing, we may have capitalized on chance findings. The generalizability of the findings is limited to women with CHD. Furthermore, we used a nonexperimental design without a control group. Second, this was a usability test of a minimal viable product with minimal contact from the research team. Third, our study was not long enough to evaluate any sustained behavior change. A randomized controlled trial with a larger sample is needed to better understand the optimal way of providing secondary prevention through digital health interventions. However, the aim of our study was to examine the usability, viability, and user requirements for developing a more comprehensive mHealth intervention for women with CHD.

### Future Directions

This usability study has encouraged us to develop a comprehensive mHealth behavior change intervention that targets PA, healthy eating, stress management, medication adherence, and smoking cessation. Such a home-based system is not intended to replace CBCR but rather to offer behavior change theory–based interventions in real time to individuals as they live their lives, particularly for those who cannot access CBCR. Evidence for the effectiveness of self-management of multiple health behaviors for improved outcomes will require a larger, randomized controlled trial of a longer duration. A pilot randomized study of the next version of HerBeat is currently underway.

Our formative evaluation of HerBeat helped us to refine our design strategy for the next trial. We plan to incorporate more provision for the user to communicate with the health coach as a group as well as individually. We have expanded the EMA surveys to target more behaviors of relevance to CV health. With feedback from the participants, we have developed many more meaningful BCTs that are deployed using decision rules in response to the participants’ responses to the EMA surveys. We have enhanced the dashboard to be more visually usable by the health coach. Finally, we have resolved some of the problems with the wearable sensor by implementing the use of a different smartwatch that has a long battery life.

### Conclusions

CV disease remains the leading cause of death worldwide. Healthy lifestyle behaviors are critical to CV health. We designed a mHealth prototype specifically for women with CHD to assist them with behavioral self-management. The participants found the prototype easy to use over 12 weeks and were receptive to setting walking goals and responding to EMA surveys. Mobile technology is an innovative and scalable approach to reducing the risk factors of CV disease, but evidence related to acceptability remains limited. Our study has contributed to the limited data on the usability of mobile apps for CV disease self-management.

## Abbreviations

BCT: behavior change technique
BP: blood pressure
CBCR: center-based cardiac rehabilitation
CHD: coronary heart disease
CV: cardiovascular
EMA: ecological momentary assessment
GUI: graphic user interface
HBCR: home-based cardiac rehabilitation
ICC: intraclass correlation coefficient
IPAQ-SF: International Physical Activity Questionnaire-Short Form
mHealth: mobile health
PA: physical activity
PHQ: Patient Health Questionnaire
PI: principal investigator
PSS: Perceived Stress Scale
REAP-S: Rapid Eating Assessment for Participants-Short Form
SE: self-efficacy
SQL: Structured Query Language
SUS: System Usability Scale

## References

[1] Balady GJ, Williams MA, Ades PA, Bittner V, Comoss P, Foody JM, Franklin B, Sanderson B, Southard D, American Heart Association Exercise‚ Cardiac Rehabilitation‚Prevention Committee‚ the Council on Clinical Cardiology, American Heart Association Council on Cardiovascular Nursing, American Heart Association Council on Epidemiology and Prevention, American Heart Association Council on Nutrition‚ Physical Activity‚Metabolism, American Association of Cardiovascular and Pulmonary Rehabilitation (2007). Core components of cardiac rehabilitation/secondary prevention programs: 2007 update: a scientific statement from the American Heart Association Exercise, Cardiac Rehabilitation, and Prevention Committee, the Council on Clinical Cardiology; the Councils on Cardiovascular Nursing, Epidemiology and Prevention, and Nutrition, Physical Activity, and Metabolism; and the American Association of Cardiovascular and Pulmonary Rehabilitation. Circulation.

[2] Smith Jr SC, Benjamin EJ, Bonow RO, Braun LT, Creager MA, Franklin BA, Gibbons RJ, Grundy SM, Hiratzka LF, Jones DW, Lloyd-Jones DM, Minissian M, Mosca L, Peterson ED, Sacco RL, Spertus J, Stein JH, Taubert KA, World Heart Federation and the Preventive Cardiovascular Nurses Association (2011). AHA/ACCF secondary prevention and risk reduction therapy for patients with coronary and other atherosclerotic vascular disease: 2011 update: a guideline from the American Heart Association and American College of Cardiology Foundation. Circulation.

[3] Anderson L, Taylor R (2014). Cardiac rehabilitation for people with heart disease: an overview of Cochrane systematic reviews. Cochrane Database Syst Rev.

[4] Anderson L, Oldridge N, Thompson D, Zwisler AD, Rees K, Martin N, Taylor RS (2016). Exercise-based cardiac rehabilitation for coronary heart disease: Cochrane systematic review and meta-analysis. J Am Coll Cardiol.

[5] Drozda Jr JJ, Messer J, Spertus J, Abramowitz B, Alexander K, Beam CT, Bonow RO, Burkiewicz JS, Crouch M, Goff DC, Hellman R, James T, King ML, Machado EA, Ortiz E, O'Toole M, Persell SD, Pines JM, Rybicki FJ, Sadwin LB, Sikkema JD, Smith PK, Torcson PJ, Wong JB (2011). ACCF/AHA/AMA-PCPI 2011 performance measures for adults with coronary artery disease and hypertension: a report of the American College of Cardiology Foundation/American Heart Association Task Force on Performance Measures and the American Medical Association-Physician Consortium for Performance Improvement. Circulation.

[6] Thomas RJ, Balady G, Banka G, Beckie TM, Chiu J, Gokak S, Ho PM, Keteyian SJ, King M, Lui K, Pack Q, Sanderson BK, Wang TY (2018). 2018 ACC/AHA clinical performance and quality measures for cardiac rehabilitation: a report of the American College of Cardiology/American Heart Association Task Force on Performance Measures. J Am Coll Cardiol.

[7] Balady GJ, Ades PA, Bittner VA, Franklin BA, Gordon NF, Thomas RJ, Tomaselli GF, Yancy CW, American Heart Association Science Advisory and Coordinating Committee (2011). Referral, enrollment, and delivery of cardiac rehabilitation/secondary prevention programs at clinical centers and beyond: a presidential advisory from the American Heart Association. Circulation.

[8] Mosca L, Benjamin E, Berra K, Bezanson JL, Dolor RJ, Lloyd-Jones DM, Newby LK, Piña IL, Roger VL, Shaw LJ, Zhao D, Beckie TM, Bushnell C, D'Armiento J, Kris-Etherton PM, Fang J, Ganiats TG, Gomes AS, Gracia CR, Haan CK, Jackson EA, Judelson DR, Kelepouris E, Lavie CJ, Moore A, Nussmeier NA, Ofili E, Oparil S, Ouyang P, Pinn VW, Sherif K, Smith SC, Sopko G, Chandra-Strobos N, Urbina EM, Vaccarino V, Wenger NK (2011). Effectiveness-based guidelines for the prevention of cardiovascular disease in women-2011 update: a guideline from the American Heart Association. Circulation.

[9] Fihn S, Gardin J, Abrams J, Berra K, Blankenship JC, Dallas AP, Douglas PS, Foody JM, Gerber TC, Hinderliter AL, King SB, Kligfield PD, Krumholz HM, Kwong RY, Lim MJ, Linderbaum JA, Mack MJ, Munger MA, Prager RL, Sabik JF, Shaw LJ, Sikkema JD, Smith CR, Smith SC, Spertus JA, Williams SV, American College of Cardiology Foundation, American Heart Association Task Force on Practice Guidelines, American College of Physicians, American Association for Thoracic Surgery, Preventive Cardiovascular Nurses Association, Society for Cardiovascular Angiography and Interventions, Society of Thoracic Surgeons (2012). 2012 ACCF/AHA/ACP/AATS/PCNA/SCAI/STS guideline for the diagnosis and management of patients with stable ischemic heart disease: a report of the American College of Cardiology Foundation/American Heart Association Task Force on practice guidelines, and the American College of Physicians, American Association for Thoracic Surgery, Preventive Cardiovascular Nurses Association, Society for Cardiovascular Angiography and Interventions, and Society of Thoracic Surgeons. J Am Coll Cardiol.

[10] Samayoa L, Grace SL, Gravely S, Scott LB, Marzolini S, Colella TJ (2014). Sex differences in cardiac rehabilitation enrollment: a meta-analysis. Can J Cardiol.

[11] Colella TJ, Gravely S, Marzolini S, Grace SL, Francis JA, Oh P, Scott LB (2015). Sex bias in referral of women to outpatient cardiac rehabilitation? A meta-analysis. Eur J Prev Cardiol.

[12] Supervía M, Medina-Inojosa JR, Yeung C, Lopez-Jimenez F, Squires RW, Pérez-Terzic CM, Brewer LC, Leth SE, Thomas RJ (2017). Cardiac rehabilitation for women: a systematic review of barriers and solutions. Mayo Clin Proc.

[13] Dreyer RP, Xu X, Zhang W, Du X, Strait KM, Bierlein M, Bucholz EM, Geda M, Fox J, D'Onofrio G, Lichtman JH, Bueno H, Spertus JA, Krumholz HM (2016). Return to work after acute myocardial infarction: comparison between young women and men. Circ Cardiovasc Qual Outcomes.

[14] Lavie CJ, Bennett A, Arena R (2017). Enhancing cardiac rehabilitation in women. J Womens Health (Larchmt).

[15] Turk-Adawi KI, Grace SL (2015). Narrative review comparing the benefits of and participation in cardiac rehabilitation in high-, middle- and low-income countries. Heart Lung Circ.

[16] Peters AE, Keeley EC (2017). Trends and predictors of participation in cardiac rehabilitation following acute myocardial infarction: data from the behavioral risk factor surveillance system. J Am Heart Assoc.

[17] Li S, Fonarow GC, Mukamal K, Xu H, Matsouaka RA, Devore AD, Bhatt DL (2018). Sex and racial disparities in cardiac rehabilitation referral at hospital discharge and gaps in long-term mortality. J Am Heart Assoc.

[18] Kotseva K, Wood D, de Bacquer D, EUROASPIRE investigators (2018). Determinants of participation and risk factor control according to attendance in cardiac rehabilitation programmes in coronary patients in Europe: EUROASPIRE IV survey. Eur J Prev Cardiol.

[19] Resurrección DM, Motrico E, Rigabert A, Rubio-Valera M, Conejo-Cerón S, Pastor L, Moreno-Peral P (2017). Barriers for nonparticipation and dropout of women in cardiac rehabilitation programs: a systematic review. J Womens Health (Larchmt).

[20] Moghei M, Turk-Adawi K, Isaranuwatchai W, Sarrafzadegan N, Oh P, Chessex C, Grace SL (2017). Cardiac rehabilitation costs. Int J Cardiol.

[21] Beckman AL, Bucholz EM, Zhang W, Xu X, Dreyer RP, Strait KM, Spertus JA, Krumholz HM, Spatz ES (2016). Sex differences in financial barriers and the relationship to recovery after acute myocardial infarction. J Am Heart Assoc.

[22] Grace SL, Gravely-Witte S, Kayaniyil S, Brual J, Suskin N, Stewart DE (2009). A multisite examination of sex differences in cardiac rehabilitation barriers by participation status. J Womens Health (Larchmt).

[23] Marzolini S, Brooks D, Oh PI (2008). Sex differences in completion of a 12-month cardiac rehabilitation programme: an analysis of 5922 women and men. Eur J Cardiovasc Prev Rehabil.

[24] Mochari H, Lee JR, Kligfield P, Mosca L (2006). Ethnic differences in barriers and referral to cardiac rehabilitation among women hospitalized with coronary heart disease. Prev Cardiol.

[25] Sanderson BK, Bittner V (2005). Women in cardiac rehabilitation: outcomes and identifying risk for dropout. Am Heart J.

[26] Beckie TM, Beckstead JW (2010). Predicting cardiac rehabilitation attendance in a gender-tailored randomized clinical trial. J Cardiopulm Rehabil Prev.

[27] Pedersen M, Overgaard D, Andersen I, Baastrup M, Egerod I (2018). Mechanisms and drivers of social inequality in phase II cardiac rehabilitation attendance: a convergent mixed methods study. J Adv Nurs.

[28] Sandesara PB, Lambert CT, Gordon NF, Fletcher GF, Franklin BA, Wenger NK, Sperling L (2015). Cardiac rehabilitation and risk reduction: time to 'rebrand and reinvigorate'. J Am Coll Cardiol.

[29] Lavie CJ, Arena R, Franklin BA (2016). Cardiac rehabilitation and healthy life-style interventions: rectifying program deficiencies to improve patient outcomes. J Am Coll Cardiol.

[30] Antypas K, Wangberg SC (2014). An internet- and mobile-based tailored intervention to enhance maintenance of physical activity after cardiac rehabilitation: short-term results of a randomized controlled trial. J Med Internet Res.

[31] Bjarnason-Wehrens B, Bott D, Benesch L, Bischoff KO, Buran-Kilian B, Gysan D, Hollenstein U, Mayer-Berger W, Wilkniss R, Sauer G (2007). Long-term results of a three-week intensive cardiac out-patient rehabilitation program in motivated patients with low social status. Clin Res Cardiol.

[32] Neubeck L, Redfern JU, Fernandez R, Briffa T, Bauman A, Freedman SB (2009). Telehealth interventions for the secondary prevention of coronary heart disease: a systematic review. Eur J Cardiovasc Prev Rehabil.

[33] Beckie TM, Beckstead JW, Schocken DD, Evans ME, Fletcher GF (2011). The effects of a tailored cardiac rehabilitation program on depressive symptoms in women: a randomized clinical trial. Int J Nurs Stud.

[34] Beckie TM, Beckstead JW (2011). The effects of a cardiac rehabilitation program tailored for women on their perceptions of health: a randomized clinical trial. J Cardiopulm Rehabil Prev.

[35] Beckie TM, Beckstead JW (2010). The effects of a cardiac rehabilitation program tailored for women on global quality of life: a randomized clinical trial. J Womens Health (Larchmt).

[36] Spring B, Moller AC, Colangelo LA, Siddique J, Roehrig M, Daviglus ML, Polak JF, Reis JP, Sidney S, Liu K (2014). Healthy lifestyle change and subclinical atherosclerosis in young adults: coronary artery risk development in young adults (CARDIA) study. Circulation.

[37] Arnett D, Blumenthal R, Albert M, Buroker AB, Goldberger ZD, Hahn EJ, Himmelfarb CD, Khera A, Lloyd-Jones D, McEvoy JW, Michos ED, Miedema MD, Muñoz D, Smith SC, Virani SS, Williams KA, Yeboah J, Ziaeian B (2019). 2019 ACC/AHA Guideline on the Primary Prevention of Cardiovascular Disease: a report of the American College of Cardiology/American Heart Association Task Force on Clinical Practice Guidelines. Circulation.

[38] Buckingham SA, Taylor RS, Jolly K, Zawada A, Dean SG, Cowie A, Norton RJ, Dalal HM (2016). Home-based versus centre-based cardiac rehabilitation: abridged Cochrane systematic review and meta-analysis. Open Heart.

[39] Anderson L, Sharp G, Norton R, Dalal H, Dean SG, Jolly K, Cowie A, Zawada A, Taylor RS (2017). Home-based versus centre-based cardiac rehabilitation. Cochrane Database Syst Rev.

[40] Tang LH, Berg SK, Christensen J, Lawaetz J, Doherty P, Taylor RS, Langberg H, Zwisler A (2017). Patients' preference for exercise setting and its influence on the health benefits gained from exercise-based cardiac rehabilitation. Int J Cardiol.

[41] Beatty AL, Truong M, Schopfer DW, Shen H, Bachmann JM, Whooley MA (2018). Geographic variation in cardiac rehabilitation participation in medicare and veterans affairs populations: opportunity for improvement. Circulation.

[42] Schopfer DW, Krishnamurthi N, Shen H, Duvernoy CS, Forman DE, Whooley MA (2018). Association of veterans health administration home-based programs with access to and participation in cardiac rehabilitation. JAMA Intern Med.

[43] Thomas RJ, Beatty AL, Beckie TM, Brewer LC, Brown TM, Forman DE, Franklin BA, Keteyian SJ, Kitzman DW, Regensteiner JG, Sanderson BK, Whooley MA (2019). Home-based cardiac rehabilitation: a scientific statement from the American Association of Cardiovascular and Pulmonary Rehabilitation, the American Heart Association, and the American College of Cardiology. J Am Coll Cardiol.

[44] Kaminsky LA, Brubaker PH, Guazzi M, Lavie CJ, Montoye AH, Sanderson BK, Savage PD (2016). Assessing physical activity as a core component in cardiac rehabilitation: a position statement of the American Association of Cardiovascular and Pulmonary Rehabilitation. J Cardiopulm Rehabil Prev.

[45] Asch DA, Muller RW, Volpp KG (2012). Automated hovering in health care-watching over the 5000 hours. N Engl J Med.

[46] Michie S, Richardson M, Johnston M, Abraham C, Francis J, Hardeman W, Eccles MP, Cane J, Wood CE (2013). The behavior change technique taxonomy (v1) of 93 hierarchically clustered techniques: building an international consensus for the reporting of behavior change interventions. Ann Behav Med.

[47] Hutchesson MJ, Rollo ME, Krukowski R, Ells L, Harvey J, Morgan PJ, Callister R, Plotnikoff R, Collins CE (2015). eHealth interventions for the prevention and treatment of overweight and obesity in adults: a systematic review with meta-analysis. Obes Rev.

[48] Lewis JR, Sauro J (2009). The Factor Structure of the System Usability Scale. Proceedings of the International Conference on Human Centered Design.

[49] Sallis JF, Pinski RB, Grossman RM, Patterson TL, Nader PR (1988). The development of self-efficacy scales for healthrelated diet and exercise behaviors. Health Educ Res.

[50] Decker JW, Dennis KE (2013). The eating habits confidence survey: reliability and validity in overweight and obese postmenopausal women. J Nurs Meas.

[51] Lorig KR, Ritter P, Stewart AL, Sobel DS, Brown BW, Bandura A, Gonzalez VM, Laurent DD, Holman HR (2001). Chronic disease self-management program: 2-year health status and health care utilization outcomes. Med Care.

[52] Cohen S, Kamarck T, Mermelstein R (1983). A global measure of perceived stress. J Health Soc Behav.

[53] Kroenke K, Spitzer RL, Williams JB (2001). The PHQ-9: validity of a brief depression severity measure. J Gen Intern Med.

[54] Fann JR, Bombardier CH, Dikmen S, Esselman P, Warms CA, Pelzer E, Rau H, Temkin N (2005). Validity of the patient health questionnaire-9 in assessing depression following traumatic brain injury. J Head Trauma Rehabil.

[55] Adewuya AO, Ola BA, Afolabi OO (2006). Validity of the patient health questionnaire (PHQ-9) as a screening tool for depression amongst Nigerian university students. J Affect Disord.

[56] Stafford L, Berk M, Jackson HJ (2007). Validity of the hospital anxiety and depression scale and patient health questionnaire-9 to screen for depression in patients with coronary artery disease. Gen Hosp Psychiatry.

[57] Segal-Isaacson CJ, Wylie-Rosett J, Gans KM (2004). Validation of a short dietary assessment questionnaire: the Rapid Eating and Activity Assessment for Participants short version (REAP-S). Diabetes Educ.

[58] Johnston CS, Bliss C, Knurick JR, Scholtz C (2018). Rapid eating assessment for participants [shortened version] scores are associated with healthy eating index-2010 scores and other indices of diet quality in healthy adult omnivores and vegetarians. Nutr J.

[59] Craig CL, Marshall AL, Sjöström M, Bauman AE, Booth ML, Ainsworth BE, Pratt M, Ekelund U, Yngve A, Sallis JF, Oja P (2003). International physical activity questionnaire: 12-country reliability and validity. Med Sci Sports Exerc.

[60] Dinger MK, Behrens TK, Han JL (2006). Validity and reliability of the international physical activity questionnaire in college students. Am J Health Educ.

[61] Wang C, Chen P, Zhuang J (2013). Validity and reliability of international physical activity questionnaire-short form in Chinese youth. Res Q Exerc Sport.

[62] Sanda B, Vistad I, Haakstad LA, Berntsen S, Sagedal LR, Lohne-Seiler H, Torstveit MK (2017). Reliability and concurrent validity of the international physical activity questionnaire short form among pregnant women. BMC Sports Sci Med Rehabil.

[63] Duncan MJ, Arbour-Nicitopoulos K, Subramanieapillai M, Remington G, Faulkner G (2017). Revisiting the international physical activity questionnaire (IPAQ): assessing physical activity among individuals with schizophrenia. Schizophr Res.

[64] American College of Sports Medicine (2010). ACSM's Guidelines For Exercise Testing And Prescription. Eighth Edition.

[65] Cornier M, Després JP, Davis N, Grossniklaus DA, Klein S, Lamarche B, Lopez-Jimenez F, Rao G, St-Onge M, Towfighi A, Poirier P, American Heart Association Obesity Committee of the Council on Nutrition, Physical Activity and Metabolism, Council on Arteriosclerosis, Thrombosis and Vascular Biology, Council on Cardiovascular Disease in the Young, Council on Cardiovascular Radiology and Intervention, Council on Cardiovascular Nursing‚ Council on Epidemiology and Prevention, Council on the Kidney in Cardiovascular Disease‚ Stroke Council (2011). Assessing adiposity: a scientific statement from the American Heart Association. Circulation.

[66] Pickering TG, Hall JE, Appel LJ, Falkner BE, Graves J, Hill MN, Jones DW, Kurtz T, Sheps SG, Roccella EJ (2005). Recommendations for blood pressure measurement in humans and experimental animals: part 1: blood pressure measurement in humans: a statement for professionals from the Subcommittee of Professional and Public Education of the American Heart Association Council on High Blood Pressure Research. Circulation.

[67] Baldwin JL, Singh H, Sittig DF, Giardina TD (2017). Patient portals and health apps: pitfalls, promises, and what one might learn from the other. Healthc (Amst).

[68] Forman DE, LaFond K, Panch T, Allsup K, Manning K, Sattelmair J (2014). Utility and efficacy of a smartphone application to enhance the learning and behavior goals of traditional cardiac rehabilitation: a feasibility study. J Cardiopulm Rehabil Prev.

[69] Johnston N, Bodegard J, Jerström S, Åkesson J, Brorsson H, Alfredsson J, Albertsson PA, Karlsson J, Varenhorst C (2016). Effects of interactive patient smartphone support app on drug adherence and lifestyle changes in myocardial infarction patients: a randomized study. Am Heart J.

[70] Widmer RJ, Allison TG, Lerman LO, Lerman A (2015). Digital health intervention as an adjunct to cardiac rehabilitation reduces cardiovascular risk factors and rehospitalizations. J Cardiovasc Transl Res.

[71] Hägglund E, Lyngå P, Frie F, Ullman B, Persson H, Melin M, Hagerman I (2015). Patient-centred home-based management of heart failure. Findings from a randomised clinical trial evaluating a tablet computer for self-care, quality of life and effects on knowledge. Scand Cardiovasc J.

[72] Bengtsson U, Kjellgren K, Hallberg I, Lindwall M, Taft C (2016). Improved blood pressure control using an interactive mobile phone support system. J Clin Hypertens (Greenwich).

[73] Layton AM, Whitworth J, Peacock J, Bartels MN, Jellen PA, Thomashow BM (2014). Feasibility and acceptability of utilizing a smartphone based application to monitor outpatient discharge instruction compliance in cardiac disease patients around discharge from hospitalization. Int J Telemed Appl.

[74] Zhou L, Bao J, Setiawan IM, Saptono A, Parmanto B (2019). The mhealth app usability questionnaire (MAUQ): development and validation study. JMIR Mhealth Uhealth.

[75] Kim S, So J (2018). How message fatigue toward health messages leads to ineffective persuasive outcomes: examining the mediating roles of reactance and inattention. J Health Commun.

[76] Davidson TL, Jones S, Roy M, Stevenson RJ (2019). The cognitive control of eating and body weight: it's more than what you 'think'. Front Psychol.

[77] Jones A, Robinson E, Duckworth J, Kersbergen I, Clarke N, Field M (2018). The effects of exposure to appetitive cues on inhibitory control: a meta-analytic investigation. Appetite.

[78] Appelhans B (2009). Neurobehavioral inhibition of reward-driven feeding: implications for dieting and obesity. Obesity (Silver Spring).

[79] Torjesen I (2018). Sixty seconds on . . . exercise. Br Med J.

[80] Althoff T, Sosič R, Hicks JL, King AC, Delp SL, Leskovec J (2017). Large-scale physical activity data reveal worldwide activity inequality. Nature.

[81] Bassett Jr DR, Toth LP, LaMunion SR, Crouter SE (2017). Step counting: a review of measurement considerations and health-related applications. Sports Med.

[82] Kraus WE, Yates T, Tuomilehto J, Sun J, Thomas L, McMurray JJ, Bethel MA, Holman RR (2018). Relationship between baseline physical activity assessed by pedometer count and new-onset diabetes in the NAVIGATOR trial. BMJ Open Diabetes Res Care.

[83] Dwyer T, Pezic A, Sun C, Cochrane J, Venn A, Srikanth V, Jones G, Shook R, Shook R, Sui X, Ortaglia A, Blair S, Ponsonby AL (2015). Objectively measured daily steps and subsequent long term all-cause mortality: the tasped prospective cohort study. PLoS One.

[84] Lee I, Shiroma EJ, Kamada M, Bassett DR, Matthews CE, Buring JE (2019). Association of step volume and intensity with all-cause mortality in older women. JAMA Intern Med.

